# Application of Protein Expression in *Mycoplasma* Study

**DOI:** 10.1155/2024/4142663

**Published:** 2024-10-14

**Authors:** Nian Xie

**Affiliations:** Faculty of Medicine, Nursing and Health Sciences, Monash University, Clayton 3168, VIC, Australia

**Keywords:** *Mycoplasma*, protein expression, protein function research, protein omics

## Abstract

*Mycoplasma* is a kind of pathogenic microorganism, and its survival and replication need to be parasitic inside the host cell. Therefore, studies on the metabolic pathway, protein composition, and biological characteristics of *Mycoplasma* require the use of protein expression techniques. In this paper, the application of protein expression in *Mycoplasma* research was reviewed, including commonly used protein expression systems, optimization strategy of protein expression, protein omics analysis, and protein function research, and the future development direction has been prospected.

## 1. Introduction

**Figure 1:**
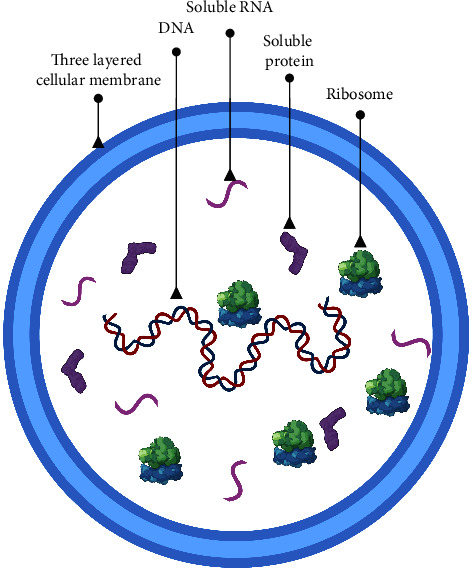
Basic structure of *Mycoplasma*.

*Mycoplasma* is a kind of pathogenic microorganism (Figure 1 illustrates the basic structure of *Mycoplasma*) that can cause a variety of diseases, such as pneumonia, influenza-like symptoms, and sexually transmitted diseases [1]. The survival and replication of *Mycoplasma* need to be parasitic inside the host cells [2], so studying its metabolic pathway, protein composition, and biological characteristics requires the use of protein expression techniques. Protein expression technology has become an indispensable tool in modern life science research, which can be used to express a variety of proteins and is further used in biological characteristic research, drug screening, and the development of diagnostic reagents [3, 4]. This article will review the application of protein expression in the *Mycoplasma* research.

## 2. *Mycoplasma* Protein Expression System

At present, *Mycoplasma* protein expression systems mainly include intracellular and extracellular expression systems [5]. The intracellular expression systems mainly include *Escherichia coli* (*E. coli*), insect-expressing cells, and mammalian cells, while the extracellular expression systems mainly include yeast and mold. Different expression systems have different advantages and disadvantages, which need to be comprehensively considered according to the specific research purpose when choosing the expression system.

In order to facilitate a comprehensive comparative analysis of the discussed protein expression systems, Table 1 provides a summary of the principal advantages and disadvantages associated with each system.

### 2.1. *E. coli* Expression System

The *E. coli* expression system is a widely used expression system with the advantages of simple operation, low cost, and high expression level [6]. *E. coli* expression systems usually use plasmid vectors to insert the target gene into *E. coli* and express it using *E. coli*'s metabolic pathway. However, the expression of *Mycoplasma* proteins in *E. coli* is often problematic because *Mycoplasma* protein is complex with numerous subcellular domains and complex modification structures, and the posttranslational modification mechanism in *E. coli* does not apply to *Mycoplasma* protein. Therefore, the application range of the *E. coli* expression system is limited [7–9].

To overcome these limitations, researchers have developed strategies to improve the *E. coli* expression system for *Mycoplasma* proteins. For example, coexpression of chaperone proteins can assist in the proper folding of *Mycoplasma* proteins and increase their solubility [10]. In addition, the use of codon optimization can enhance the expression levels of *Mycoplasma* proteins in *E. coli* by adapting the codon usage to that of the host. Furthermore, modifications to the culture conditions, such as adjusting temperature and inducer concentration, can also help improve the expression of *Mycoplasma* proteins in *E. coli* [11]. Despite these efforts, the *E. coli* expression system might still not be ideal for all *Mycoplasma* proteins, particularly due to its limited ability to handle posttranslational modifications. For instance, proteins such as P1 adhesin and multiple banded antigen (MBA), which require glycosylation or contain multiple disulfide bonds, present significant challenges when expressed in *E. coli*.

### 2.2. Insect Cell Expression System

The insect cell expression system is a common expression system with the advantages of efficient expression of heterologous protein and posttranslational modification similar to mammalian cells [12]. Two common systems for expressing insect cells are Sf9 and Sf21. Both cells are derived from ovarian tissue in Hyphantria and can be used for large-scale *Mycoplasma* protein expression [13]. The baculovirus expression vector system (BEVS) is the most widely used method for protein expression in insect cells. It allows for high-level protein expression and offers the advantage of eukaryotic posttranslational modifications.

However, the insect cell expression system also faces some challenges. For example, the baculovirus infection process can be time-consuming, and the virus may be unstable, leading to a decrease in protein expression levels [14]. In addition, protein expression may be hampered by proteolytic degradation, necessitating the use of protease inhibitors or the coexpression of protease-resistant proteins [15]. To improve the insect cell expression system for *Mycoplasma* proteins, researchers have employed strategies such as optimizing the baculovirus vector design, utilizing fusion tags to enhance protein solubility, and modifying culture conditions to increase protein yield and stability.

### 2.3. Mammalian Cell Expression System

The mammalian cell expression system is one of the most suitable expression systems for *Mycoplasma* protein expression. The posttranslational modifications in mammalian cell systems can be similar to those of *Mycoplasma* protein and more accurately mimic the biological behavior of *Mycoplasma* protein in host cells [9]. In addition, the mammalian cell expression system is more suitable for the expression of complex proteins. However, mammalian cell expression systems are costly and require a long period of expression.

Common mammalian cell lines used for protein expression include Chinese hamster ovary (CHO) cells, human embryonic kidney (HEK) 293 cells, and baby hamster kidney (BHK) cells [16]. These cell lines offer advantages such as efficient protein expression, correct folding, and appropriate posttranslational modifications for *Mycoplasma* proteins. Despite their benefits, mammalian cell expression systems can still present challenges, including low protein yields, complex culture conditions, and the potential risk of viral contamination.

To overcome these challenges, researchers have developed various strategies to optimize mammalian cell expression systems for *Mycoplasma* proteins [17]. These include the use of strong and inducible promoters, the incorporation of specific signal peptides to enhance secretion, and the use of gene amplification to increase protein expression levels. Moreover, advances in cell culture techniques, such as the development of serum-free and chemically defined media, have improved the scalability and reproducibility of mammalian cell expression systems. Furthermore, the utilization of transient expression systems allows for rapid protein production, which can be advantageous for the expression of *Mycoplasma* proteins that may be toxic to host cells.

### 2.4. Yeast Expression System

Yeast expression systems, such as *Saccharomyces cerevisiae* and *Pichia pastoris*, have been widely used for the production of recombinant proteins, including *Mycoplasma* proteins. *S. cerevisiae* is a well-established model organism with a fully sequenced genome and a wealth of genetic tools available for manipulation. *P. pastoris*, on the other hand, offers advantages such as high cell density cultivation, strong methanol-inducible promoters, and the ability to perform complex glycosylation [18].

To enhance the expression of *Mycoplasma* proteins in yeast systems, researchers have employed various strategies, including codon optimization, utilization of strong promoters, and optimization of culture conditions [19]. In addition, coexpression of chaperone proteins or the fusion of target proteins with solubility-enhancing partners can improve protein solubility and folding. Despite these improvements, the lower protein yields and potential differences in glycosylation patterns compared to mammalian cells may limit the applicability of yeast expression systems for certain *Mycoplasma* proteins.

### 2.5. Mold Expression System

Mold expression systems, such as *Aspergillus niger* and *Trichoderma reesei*, are widely used for the production of recombinant proteins, including enzymes, antibodies, and vaccines [20]. These filamentous fungi are known for their ability to secrete large quantities of proteins into the culture medium, which simplifies downstream purification processes. In addition, mold expression systems are capable of performing many posttranslational modifications, including glycosylation and disulfide bond formation.

Optimization strategies for mold expression systems include the use of strong and inducible promoters, codon optimization, and the utilization of fusion partners to improve protein stability and secretion. Furthermore, advances in genetic engineering techniques, such as CRISPR/Cas9, allow for the targeted modification of mold expression hosts to improve protein production and secretion [21]. However, the potential heterogeneity of the expressed protein and the presence of fungal proteases in the culture medium may necessitate additional purification and modification steps to obtain the desired protein product.

### 2.6. Extracellular Membrane Vesicle (EV) Expression System

EVs are vesicles with membrane structures secreted by cells, and the size ranges from 20 nm to 5 *μ*m, which can contain a variety of bioactive molecules, such as protein, nucleic acid, and lipid substances. It was also used in the study of *Mycoplasma* protein as a novel protein expression and delivery system [22].

Vesicles can be released from host cells and transferred within other cells, thus being considered a potential drug delivery pathway. Because of their specificity, stability, high abundance, and traceability, EVs have become a hot research object in the fields of intercellular communication, disease diagnosis, and treatment. Recent studies have shown that EVs can play a critical role in various biological processes, including immune response modulation, cell-to-cell communication, and even the progression of some diseases such as cancer. Due to their unique characteristics, EVs have gained significant attention as a potential alternative for delivering therapeutic molecules, including proteins, peptides, and small interfering RNA (siRNA).

In recent years, researchers have begun to explore the potential of EVs as a carrier for the expression of the exogenous protein [23]. In the *Mycoplasma* research, some researchers also began to use EVs as expression vectors for the expression of various *Mycoplasma* proteins. A study has shown that MOMP-EVs can be successfully expressed by cloning *Mycoplasma* MOMP-protein into an EV expression vector. These MOMP-EVs can be taken up by human lung epithelial cells (A549) and mouse macrophages (RAW264.7) and can induce an immune response, indicating that this technology has the potential to be applied to the *Mycoplasma* research [24]. However, the expression efficiency of the EV expression system is still limited to a certain extent. To overcome this limitation, researchers have been investigating methods to improve EV loading efficiency, such as engineering the producer cells or modifying the target proteins. As the encapsulation mechanism of the EVs is closely related to the expression regulation mechanism of the cells themselves, an in-depth understanding of the cell secretion pathway and membrane transport mechanism is required to achieve high-efficiency expression in an EV vector so that reasonable optimization and adjustment can be performed during the design and construction of the expression vector [25].

## 3. Application of *Mycoplasma* Protein Expression in Research

### 3.1. Basic Research

The study of *Mycoplasma* protein expression is crucial for understanding various aspects of *Mycoplasma* biology, including pathogenesis, host-pathogen interactions, and immune evasion strategies. By expressing and characterizing various *Mycoplasma* proteins, researchers can investigate their roles in cellular processes such as adhesion, invasion, and immune modulation.

Moreover, the expression of *Mycoplasma* proteins enables the study of their three-dimensional structures using techniques such as X-ray crystallography and cryo-electron microscopy [26]. Structural information provides insights into the molecular mechanisms underlying protein function and can guide the rational design of therapeutic interventions. Furthermore, *Mycoplasma* protein expression facilitates the identification and characterization of protein-protein interactions, both within the *Mycoplasma* and between the *Mycoplasma* and host cells [27]. These interactions are critical for understanding the complex interplay between *Mycoplasma* and their host cells, which may aid in the development of targeted therapies and vaccines.

### 3.2. Diagnosis and Treatment

*Mycoplasma* infections pose a significant threat to human health due to the microorganism's ability to evade the immune system and persist within host cells. For example, *Mycoplasma* pneumoniae is known to cause a range of respiratory diseases from mild tracheobronchitis to severe pneumonia [1, 27], particularly in immunocompromised individuals, the elderly, and young children. In addition to respiratory infections, *Mycoplasma* species are also associated with chronic inflammatory diseases such as asthma [28] and chronic obstructive pulmonary disease (COPD) [29, 30]. In rare cases, these infections can lead to systemic complications such as septicemia [31] and encephalitis [32]. A major concern with *Mycoplasma* infections is their resistance to traditional antibiotics, particularly beta-lactams [33], as these organisms lack a cell wall. Coupled with their ability to form biofilms and establish persistent infections, this resistance underscores the urgent need for novel therapeutic strategies, including the development of vaccines and targeted antimicrobials.

*Mycoplasma* protein expression plays a crucial role in the development of diagnostic tools and therapeutic strategies for *Mycoplasma* infections. The ability to express and purify immunogenic *Mycoplasma* proteins allows for the production of specific monoclonal and polyclonal antibodies, which are essential for various diagnostic assays, such as enzyme-linked immunosorbent assays (ELISA) and immunofluorescence assays. Moreover, *Mycoplasma* protein expression aids in the identification of novel biomarkers, which are critical for the early detection and monitoring of *Mycoplasma* infections [34]. To further clarify the significance of *Mycoplasma* protein expression technology in disease diagnosis, these details are summarized in Table 2.

*Mycoplasma* protein expression plays a crucial role in the development of new drugs and vaccines aimed at combating these pathogens. By expressing and characterizing key *Mycoplasma* proteins, researchers are able to identify potential drug targets and screen for inhibitors with antimicrobial properties. In addition, the expression of these proteins facilitates the assessment of their immunogenicity and protective efficacy, making them promising candidates for vaccine development [40]. Effective vaccines against *Mycoplasma* infections could significantly reduce the global disease burden and help prevent the emergence of antibiotic-resistant strains. The applications of *Mycoplasma* protein expression technology in disease treatment are summarized in Table 3.

## 4. Problems and Challenges in Protein Expression of *Mycoplasma*

The expression of *Mycoplasma* proteins faces various challenges, including difficulties in protein folding, posttranslational modifications, and purification.  Table 4 summarizes the key factors and challenges associated with these proteins.

### 4.1. Low Protein Expression

Low protein expression levels may arise due to the complex structure and modifications of *Mycoplasma* proteins. To address this issue, researchers can optimize the expression system by adjusting factors such as the choice of host organism, promoter strength, and codon usage. This might involve selecting a host organism that is better suited for the expression of *Mycoplasma* proteins or utilizing strong promoters and codon-optimized genes to enhance transcription and translation efficiency.

Another strategy to improve protein expression is to employ molecular chaperones or fusion partners. Molecular chaperones can facilitate proper folding and prevent protein aggregation, thereby increasing protein solubility and stability. Fusion partners, on the other hand, can be used to enhance protein expression by promoting solubility, stability, or proper localization within the host cell. Common fusion partners include maltose-binding protein (MBP), glutathione S-transferase (GST), and thioredoxin (Trx), which can be removed postexpression through the use of specific protease cleavage sites [49].

In some cases, optimizing expression conditions, such as temperature, induction time, and media composition, can also contribute to increased protein expression levels [50]. Furthermore, employing high–throughput screening methods can help to identify optimal conditions more efficiently by simultaneously testing multiple expression parameters. By considering these strategies, researchers can work to overcome the challenges of low protein expression in *Mycoplasma* studies and enhance the overall success of protein expression project.

### 4.2. Protein Folding and Modification Problems

Proper folding and posttranslational modifications of *Mycoplasma* proteins are critical for their functionality and stability. Challenges in this area may result from the inability of the expression system to correctly fold and modify the expressed protein. To overcome these obstacles, researchers can optimize the expression system by adding chaperone proteins, coexpressing modifying enzymes, or adjusting expression conditions such as temperature, induction time, and media composition.

Chaperone proteins, such as GroEL-GroES and DnaK-DnaJ-GrpE systems, can assist in the folding process and prevent aggregation of nascent polypeptides, thereby increasing the solubility and proper folding of the target protein [51]. Coexpression of modifying enzymes, such as glycosyltransferases and protein disulfide isomerases, can facilitate correct posttranslational modifications, which are essential for protein function and stability.

Adjusting expression conditions can also have a significant impact on protein folding and modification. For example, lowering the expression temperature can slow down the translation rate, allowing more time for the protein to fold correctly and acquire necessary modifications. Induction time and media composition can also be optimized to ensure that the host organism has sufficient nutrients and energy to support proper folding and modification processes [52].

### 4.3. Purification of Protein

The purification of *Mycoplasma* proteins also presents challenges due to their complex structures and modifications [53]. Conventional protein purification methods may not be sufficient to obtain high-purity proteins in these cases. To address this issue, researchers can optimize the purification process by employing various strategies. These may include the use of specific buffers to maintain protein stability and solubility, the selection of appropriate affinity chromatography columns to improve binding specificity, and the application of advanced purification techniques such as size exclusion chromatography, ion exchange chromatography, or hydrophobic interaction chromatography [54]. These optimizations can help to obtain high-purity *Mycoplasma* proteins that are suitable for downstream applications in research and biotechnology.

## 5. Conclusion and Outlook

In conclusion, *Mycoplasma* protein expression technology holds significant promise for advancing our understanding of *Mycoplasma* biology and improving the diagnosis and treatment of *Mycoplasma* infections. Through the expression and purification of *Mycoplasma* proteins, researchers can elucidate the roles and mechanisms of these proteins in *Mycoplasma* pathogenesis, host-pathogen interactions, and cellular processes. This knowledge can pave the way for the development of novel diagnostic tools, therapeutic interventions, and prevention strategies for *Mycoplasma*-associated diseases.

Despite its potential, *Mycoplasma* protein expression technology still faces numerous challenges, including low expression levels, protein folding and modification issues, and purification difficulties. Overcoming these obstacles requires the optimization of expression systems and purification processes, as well as the development of innovative strategies for protein engineering and design. Continued advancements in molecular biology, biotechnology, and bioinformatics will enable researchers to address these challenges more effectively and efficiently.

In the future, as technology continues to progress, *Mycoplasma* protein expression technology is expected to play an increasingly important role in the research, diagnosis, and treatment of *Mycoplasma* infections. This will lead to a better understanding of the molecular mechanisms underlying *Mycoplasma* biology, facilitating the identification of potential drug targets and biomarkers for disease monitoring. Moreover, the development of novel expression systems, such as extracellular vesicle–based platforms, may further expand the applications of *Mycoplasma* protein expression technology in areas such as vaccine development and targeted drug delivery.

## Figures and Tables

**Table 1:** Comparative analysis of different protein expression systems for *Mycoplasma* research.

Expression system	Advantages	Disadvantages
*E. coli*	Facilitates high-level expression at low operational costs and is characterized by straightforward manipulative procedures	Constrained by its limited capacity for posttranslational modifications and challenges in folding and solubility of complex proteins
Insect cell systems	Capable of efficient heterologous protein expression and supports eukaryotic-like posttranslational modifications	The baculovirus-based processes are labor-intensive, and the system is prone to viral instability and proteolytic degradation
Mammalian cell systems	Optimal for expression of complex proteins, ensuring correct folding and authentic posttranslational modifications	High operational costs, intricate culture conditions, relatively low protein yields, and potential for viral contamination
Yeast systems	High–cell density cultivation, robust inducible promoters, and the ability to perform complex glycosylation	Protein yields may be lower compared to mammalian systems, with potential discrepancies in glycosylation profiles
Mold systems	Exceptional secretion capabilities and can execute a range of posttranslational modifications including glycosylation.	Challenges include protein heterogeneity and the presence of fungal proteases, complicating downstream purification processes
EVs	Offer a novel platform for targeted delivery, encapsulating a diverse array of bioactive molecules	Expression efficiency remains suboptimal, requiring sophisticated engineering to enhance cell secretion pathways

**Table 2:** Applications of *Mycoplasma* protein expression in disease diagnosis.

Application	Description	Significance
Rapid point-of-care tests [35]	Development of lateral flow immunoassays (LFIAs) for quick detection of *Mycoplasma* in respiratory samples	Enables rapid and accurate diagnosis in clinical settings
Multiplex qPCR assay [36]	Development of qPCR assays capable of simultaneously detecting multiple *Mycoplasma* species	Allows comprehensive screening and differentiation between species
Advanced imaging techniques [37]	Development of imaging agents for PET and SPECT scans using *Mycoplasma* proteins conjugated with radioactive isotopes	Provides noninvasive diagnosis and monitoring of infection
Automated high–throughput screening [38]	Automation of ELISA and other immunoassays for large-scale screening in hospital laboratories	Increases the speed and accuracy of *Mycoplasma* detection
NGS-based diagnostics [39]	Design of probes and primers for NGS assays to enhance specificity and sensitivity in genomic analysis	Enables comprehensive genomic analysis, including resistance markers

**Table 3:** Applications and significance of *Mycoplasma* protein utilization in therapeutic development.

Application	Description	Significance
Monoclonal antibody development [41]	Production of specific monoclonal antibodies targeting *Mycoplasma* surface proteins to neutralize the pathogen	Enhances treatment options, especially for antibiotic-resistant *Mycoplasma* infections
Targeted drug delivery [42]	Development of drug delivery systems using *Mycoplasma* proteins to specifically target infected cells	Increases the efficacy of treatments while minimizing side effects
Antibiofilm strategies [43]	Identification and targeting of biofilm-associated proteins expressed by *Mycoplasma* to disrupt biofilm formation	Improves the effectiveness of antibiotics by breaking down biofilms that protect pathogen cells
New drug targets [44]	Identification of potential drug targets through the expression of essential *Mycoplasma* proteins	Facilitates the screening and development of new antimicrobial agents
Vaccine development [45]	Evaluation of expressed *Mycoplasma* proteins for their immunogenicity and protective efficacy as vaccine candidates	Aids in the creation of effective vaccines to reduce *Mycoplasma*'s global disease burden

**Table 4:** Key factors and challenges in *Mycoplasma* protein expression.

Protein	Gene	Function	Challenges
Adhesin P1 [46]	mgpA	Involved in *Mycoplasma*'s adherence to host cells	Requires glycosylation and disulfide bonds, difficult to express in *E. coli* due to complex posttranslational modifications
MBA [47]	mba	Surface antigen plays a role in immune evasion	Complex structures with multiple disulfide bonds, challenging to express in *E. coli* due to the need for proper folding and posttranslational modifications
Chaperonin GroEL [48]	groEL	Together with its cochaperonin GroES, it plays an essential role in assisting protein folding	GroEL functions as a complex oligomer, typically forming a tetradecameric structure. Incorrect oligomerization during expression can result in nonfunctional or unstable protein

## Data Availability

Data sharing is not applicable to this article as no datasets were generated or analyzed during the current study.
